# Culture Negative* Listeria monocytogenes* Meningitis Resulting in Hydrocephalus and Severe Neurological Sequelae in a Previously Healthy Immunocompetent Man with Penicillin Allergy

**DOI:** 10.1155/2015/248302

**Published:** 2015-12-01

**Authors:** Shahin Gaini, Gunn Hege Karlsen, Anirban Nandy, Heidi Madsen, Debes Hammershaimb Christiansen, Sanna á Borg

**Affiliations:** ^1^Medical Department, Infectious Diseases Division, National Hospital of the Faroe Islands, 100 Tórshavn, Faroe Islands; ^2^Infectious Diseases Research Unit, Odense University Hospital and University of Southern Denmark, 5000 Odense, Denmark; ^3^Department of Science and Technology, University of the Faroe Islands, 100 Tórshavn, Faroe Islands; ^4^National Reference Laboratory for Fish and Animal Diseases, Faroese Food Security Agency, 100 Tórshavn, Faroe Islands

## Abstract

A previously healthy 74-year-old Caucasian man with penicillin allergy was admitted with evolving headache, confusion, fever, and neck stiffness. Treatment for bacterial meningitis with dexamethasone and monotherapy ceftriaxone was started. The cerebrospinal fluid showed negative microscopy for bacteria, no bacterial growth, and negative polymerase chain reaction for bacterial DNA. The patient developed hydrocephalus on a second CT scan of the brain on the 5th day of admission. An external ventricular catheter was inserted and* Listeria monocytogenes* grew in the cerebrospinal fluid from the catheter. The patient had severe neurological sequelae. This case report emphasises the importance of covering empirically for* Listeria monocytogenes* in all patients with penicillin allergy with suspected bacterial meningitis. The case also shows that it is possible to have significant infection and inflammation even with negative microscopy, negative cultures, and negative broad range polymerase chain reaction in cases of* Listeria* meningitis. Follow-up spinal taps can be necessary to detect the presence of* Listeria monocytogenes*.

## 1. Introduction

*Listeria monocytogenes* (LM) meningitis is a rare disease entity with an estimated incidence of 0.03–0.2 cases/100.000 people/year [1, 2]. The disease is mainly transmitted by contaminated food and has been associated with newborn infants, pregnant women, and patients with comorbidity, to elderly and to immunosuppressed individuals [3–6].* Listeria* meningitis can be difficult to diagnose because of no optimal sensitivities in diagnostic tests of the cerebrospinal fluid (CSF) and blood cultures [7, 8]. The patient reported in this case report illustrates very well the clinical dilemmas in this serious condition, where even modern laboratory analyses showed failing sensitivities in a patient with penicillin allergy, not covered for* Listeria* infection up-front at the time of admission.

## 2. Case Presentation

**Figure 1:**
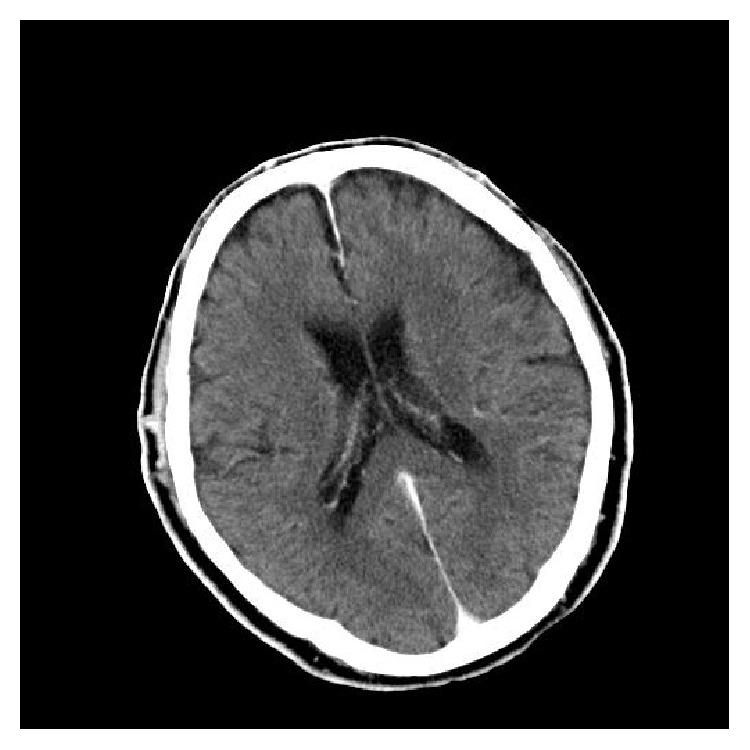
CT of the brain with intravenous contrast on the first day of admission showing no intracranial pathology, no hydrocephalus, and no brain abscess formation.

**Figure 2:**
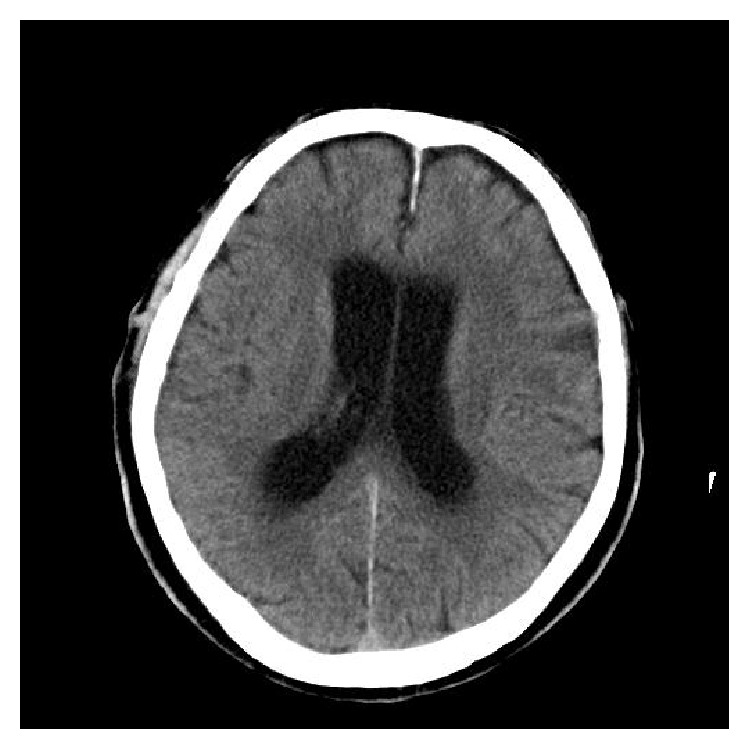
CT of the brain without intravenous contrast on the fifth day of admission showing development of a communicating hydrocephalus.

**Figure 3:**
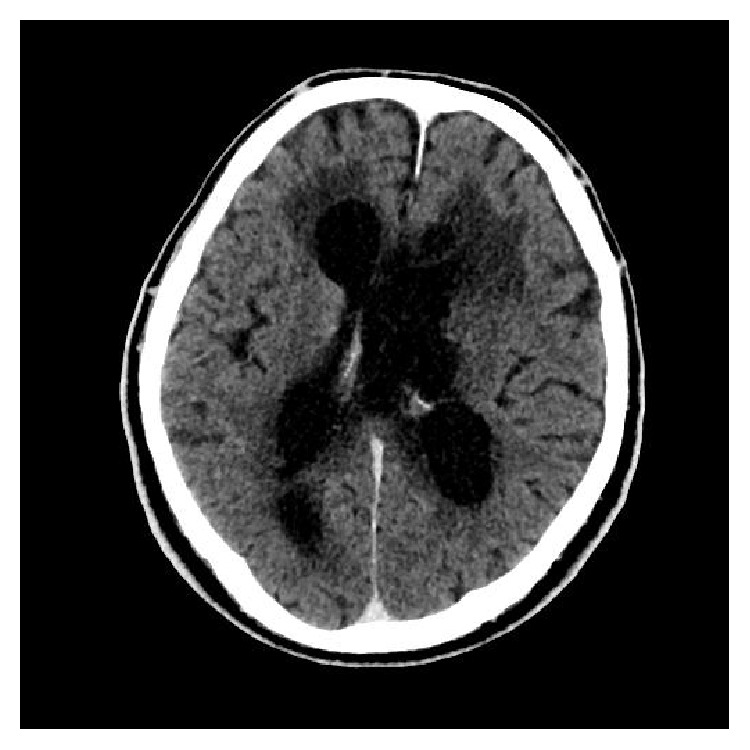
CT of the brain with intravenous contrast 6 months after admission showing progression of hydrocephalus.

A 74-year-old Caucasian immunocompetent man was admitted with an anamnesis of just 24 hours with evolving headache, fever, and confusion. The patient was known for having an uncomplicated essential hypertension treated with amlodipine; epilepsy treated with lamotrigine and no convulsions for many years; no previous hospital admissions. He was fit and physically active as a hobby farmer and as an active mountain walker. At the time of admission he had a Glasgow Coma Scale (GCS) score of 14 points, temperature of 40 degrees Celsius, normal vital parameters, petechia on both legs, and neck stiffness. He had no focal neurological deficits and no convulsions. Blood chemistry showed leukocytes of 14.1 × 10^9^/L, neutrophils of 12.2 × 10^9^/L, creatinine of 119 micromol/L, INR of 1.2, blood glucose of 7.7 mmol/L, and a C-reactive protein (CRP) of 131 mg/L (reference value: <3 mg/L). A spinal tap showed turbid CSF with pleocytosis of 1877 × 10^6^/L, 78% of neutrophils, CSF protein of 1.9 g/L, CSF glucose of 3.7 mmol/L, and CSF lactate of 5.2 mmol/L (reference value: 0.9–2.8 micromol/L). Gram staining of the CSF was negative for bacteria. After the spinal tap the patient was started promptly on treatment with intravenous dexamethasone 10 mg four times a day for 4 days and with intravenous ceftriaxone 4 g once daily, according to our guidelines in 2011 for empirical treatment of bacterial meningitis in patients with penicillin allergy. According to our guidelines patients without penicillin allergy should be treated with combination antibiotic treatment of ceftriaxone and benzylpenicillin or ampicillin (as coverage for possible* Listeria* meningitis). During the first day of admission the GCS score fell to under 12 points and a CT of the brain was performed with no signs of hydrocephalus or other complications to bacterial meningitis (Figure 1). Because neither the CSF nor the blood cultures showed any growth on the second day of admission, CSF was sent to the National Reference Laboratory of Microbiology in Copenhagen, Denmark (Statens Serum Institut), for extensive polymerase chain reaction (PCR) analyses for bacterial and viral pathogens. These PCR examinations of the CSF from the day of admission were negative for herpes simplex virus, varicella zoster virus, Cytomegalovirus, Epstein-Barr virus, Enterovirus, and* Mycoplasma pneumoniae*, specific PCR for* Streptococcus pneumoniae*, specific PCR for* Neisseria meningitidis*, and a broad range 16sRNA PCR for bacterial DNA. A urine sample was also negative for pneumococcal urine antigen. At the same time treatment was intensified with intravenous aciclovir 750 mg three times a day. On the third and fourth day of admission the patient seemed to improve a little bit clinically, with rising GCS score to 14, falling levels of CRP, but still with temperature over 40 degrees Celsius despite maximum dose of antipyretic paracetamol (4 g/day). On the fifth day of admission his clinical state deteriorated with falling GCS score under 10 and eye deviation to the left side but otherwise without neurological deficits. A new CT of the brain on the fifth day of admission now showed development of a communicating hydrocephalus (Figure 2). The patient was changed from ceftriaxone to intravenous meropenem 2 g three times daily on the indication of clinical failure on ceftriaxone treatment. The patient was transferred with ambulance airplane from the Faroe Islands to Copenhagen, Denmark, where an external ventricular catheter was inserted the same evening. CSF from this catheter showed fast growth of LM with normal resistance patterns and as expected resistance to cephalosporins. The* Listeria* strain was typed at the Faroese Food Security Agency as LM serotype 1/2a. After the identification of LM in the CSF, aciclovir was discontinued, and the patient was treated with meropenem for 6 weeks. A small brain abscess formation in relation to the external ventricular catheter was the reason for the long meropenem treatment. This small brain abscess formation was interpreted as a complication to the external ventricular catheter treatment. The patient was treated at the intensive care unit (ICU) for 5 weeks, four of these weeks on ventilator. During the stay at the ICU the patient had to have inserted external ventricular catheter three times. When the antibiotic treatment finished, the patient had severe neurological sequelae with tetraparesis, convulsions, and very low cognitive functions. A control CT of the brain 7 months after admission showed severe sequelae in the brain structure (Figure 3). The patient was admitted to a normal medical ward for one whole year, before being sent to a nursing home, but died shortly after. There was no indication of how the patient was infected with LM and no food investigation was done relating to this case.

## 3. Discussion

LM meningitis is a very serious disease with an estimated case fatality rate of 17–24% [6, 8]. Data indicate a rise in the incidence of severe infections with LM [1, 2]. LM infections have previously been associated with the extreme of ages, involving newborns and the elderly, and also associated with significant comorbidity, immunosuppression, and pregnancy [3–5]. Up to 30–40% of LM meningitis cases are occurring in immunocompetent elderly patients [2].

From the literature it is known that only approx. 10–30% of Gram stains of CSF are positive in LM meningitis [7]. Cultures of the CSF do not have an optimal sensitivity with positive cultures in 83% of patients with LM meningitis [8]. Finally blood cultures are positive in only 46–64% of LM meningitis patients [6, 8]. However in most cases one or more of the diagnostic modalities (CSF Gram stain, CSF culture, and blood cultures) are positive for LM. Reports have also been on patients diagnosed with molecular methods in the form of PCR, identifying DNA from LM meningitis [3, 9]. The literature has previously reported the need of reevaluation with follow-up spinal tap of initially microscopy/culture negative patients [10]. Follow-up spinal taps have previously identified LM meningitis like in our case [10].

Hydrocephalus is a known potential complication of bacterial meningitis in approx. 5% of cases and has been associated with LM meningitis [6, 8, 11]. The occurrence of hydrocephalus in LM meningitis cases has been reported to be 14-15% [6–8]. The prognosis of hydrocephalus patients with LM is poor and showed an unfavourable outcome in 100% of patients in a Dutch study with LM meningitis and hydrocephalus [11]. In this study all four patients with the combination of hydrocephalus and meningitis, treated with external ventricular catheter, had a poor outcome, with three fatal cases and one with severe neurological sequelae [11]. A Spanish observational study on LM meningitis showed that the combination of LM meningitis and hydrocephalus had a poor outcome with a mortality of 43% of the patients [8]. In the same study the mortality rate in patients with LM meningitis and hydrocephalus treated with external ventricular catheter was 29% [8].

Third-generation cephalosporins are the backbone of empirical treatment of bacterial meningitis, until the treatment can be adjusted to the identified pathogen and the resistance pattern [12]. Third-generation cephalosporins cover the most common pathogens,* Streptococcus pneumoniae* and* Neisseria meningitides*, but also other streptococci, MSSA* Staphylococcus aureus* and enterobacteria [12]. In countries with high level of cephalosporin-resistant pneumococci, MRSA* Staphylococcus aureus*, or/and ESBL enterobacteria, vancomycin or/and meropenem can be needed in empirical regimes for bacterial meningitis [12]. LM is an important exception and resistant to cephalosporins [12]. Therefore it is common in empirical antibiotic regimes to cover for the possibility of LM with the addition of ampicillin or amoxicillin to the treatment with a third-generation cephalosporin [12]. In Scandinavia benzylpenicillin is often used to cover for* Listeria monocytogenes*, as add-on drug to the backbone treatment with a third-generation cephalosporin [13]. Our guidelines did not cover for LM meningitis in cases with penicillin allergy in 2011, when our case occurred. In the new national Danish guidelines for bacterial meningitis, meropenem is recommended for patients with penicillin allergy [14]. The experience in using meropenem in LM is limited, but data suggest it can be used [15, 16]. Sulfamethoxazole/trimethoprim has for a longer time been used as an alternative non-beta-lactam antibiotic to ampicillin, amoxicillin, or benzylpenicillin, in patients with penicillin allergy [12]. In many countries gentamicin is added to ampicillin, amoxicillin, or benzylpenicillin to have a synergistic effect in culture proven infection with LM [12]. However studies have also indicated that the use of gentamicin in LM meningitis could increase kidney damage and mortality, so the role of gentamicin in LM meningitis is pending [17]. The patient in this case report was not covered for LM for 4 days, before being switched over from ceftriaxone to meropenem. The clinical improvement the first two days, with falling CRP, may be related to the four-day treatment with dexamethasone. Most cases of LM meningitis will be positive in one or more of the following diagnostic test methods/samples within 24–48 hours: CSF Gram stain, CSF cultures, or the blood cultures. A positive early test for LM in our case would have guided our clinicians in an earlier stage towards the diagnosis of LM and a relevant antibiotic change could have been done. The combination of penicillin allergy and therefore avoidance of benzylpenicillin (or ampicillin or amoxicillin) covering for LM up-front in our patient, combined with negative CSF Gram stain, negative CSF cultures, negative blood cultures, and negative CSF broad range PCR for bacterial DNA, was very unfortunate. Our patient demonstrates the weaknesses of even modern sensitive molecular methods like broad range PCR in diagnosing LM meningitis. Even if our patient showed significant clinical and biochemical inflammation of the central nervous system up-front at admission, LM could not be detected in the CSF sample taken before administration of antibiotics. It is possible that the inoculum of LM in our patient was extremely small and therefore undetectable in microscopy, in culturing of the CSF, in blood cultures, and in our broad range 16sRNA PCR for bacterial DNA. Still this possibly small inoculum seemed to provoke a significant inflammatory and clinical response with significant clinical symptoms and clinical findings lasting only 24 hours before admission and with negative culture of the first spinal tap. It is possible that the potent dexamethasone immunosuppressive treatment for four days, following standard protocol for bacterial meningitis, optimised conditions for growth of LM in the CSF over the next 5 days. On top of this, the patient developed hydrocephalus during the first 5 days after admission, resulting in a long stay at the ICU, intubated in ventilator with insertion of external ventricular catheters three times by the neurosurgeons. Clinically and documented with CT of the brain, 7 months after the admission he had severe neurological sequelae and tetraparesis.

A rare and severe manifestation of neuroinfection with LM is rhombencephalitis (RE), involving the brain stem and the cerebellum, normally visualised with MRI of the brain [18]. No MRI of the brain was performed in our case, and therefore we cannot exclude a possible presence of RE in our patient. LM RE usually follows a biphasic time course, with first a flu-like prodrome in up to 15 days, before severe meningitis and/or encephalitis symptoms occur, resulting in acute hospital admission [18]. LM RE occurs in younger patients than classical LM meningitis/encephalitis and occurs also in immunocompetent patients [18]. Unilateral cranial nerve deficits are almost always present in LM RE [18]. The patient described in this case report was elderly, although immunocompetent. He had no anamnesis of a flu-like prodrome before his admission and he had no cranial nerve deficits. His symptoms and his anamnesis were therefore not typical of LM RE, but no MRI of the brain was performed in the acute phase of his LM meningitis, and therefore we cannot rule out LM RE definitely.

In conclusion we present a patient with up-front culture negative LM meningitis, combined with the presence of penicillin allergy, and therefore lacking antibiotic coverage for LM the first 4 days of admission. The patient developed hydrocephalus and severe neurological sequelae and died after one year. This case report emphasises the importance of antibiotic coverage for LM in all patients with suspected bacterial meningitis, including those with penicillin allergy. It also emphasises the need to reevaluate the diagnosis with follow-up spinal taps to detect possible evolving LM infection in patients with suspected bacterial meningitis and lacking clinical response on the empirical treatment and at the same time microscopy/culture negative CSF, culture negative blood, and negative broad range 16sRNA examinations for bacterial DNA.
